# No evidence for the benefit of PPIs in the treatment of acute pancreatitis: a systematic review and meta-analysis

**DOI:** 10.1038/s41598-023-29939-5

**Published:** 2023-02-16

**Authors:** István László Horváth, Stefania Bunduc, Balázs Hankó, Dénes Kleiner, Alexandra Demcsák, Bence Szabó, Péter Hegyi, Dezső Csupor

**Affiliations:** 1 Centre for Translational Medicine, Semmelweis University, Üllői út 26, 1085 Budapest, Hungary; 2 University Pharmacy Department of Pharmacy Administration, Hőgyes Endre utca 7-9, 1092 Budapest, Hungary; 3 Division of Pancreatic Diseases, Heart and Vascular Center, Semmelweis University, Baross út 22-24, 1085 Budapest, Hungary; 4 Carol Davila University of Medicine and Pharmacy, Dionisie Lupu Street 37, 020021 Bucharest, Romania; 5 Fundeni Clinical Institute, Fundeni Street 258, 022328 Bucharest, Romania; 6 Department of Surgery, University of California Los Angeles, 675 Charles E Young Dr. S MRL 2220, Los Angeles, CA 90095 USA; 7 Institute for Translational Medicine, Medical School, University of Pécs, Szigeti út 12, 7624 Pécs, Hungary; 8 Institute of Clinical Pharmacy, University of Szeged, Szikra utca 8, 6725 Szeged, Hungary

**Keywords:** Gastroenterology, Translational research

## Abstract

Although current guidelines do not recommend the use of proton pump inhibitors (PPIs) in the standard of care of acute pancreatitis (AP), they are often prescribed in clinical practice, mainly for ulcer stress prophylaxis. In this systematic review and meta-analysis we evaluated the association between the use of PPIs in the management of AP and various clinical outcomes. We conducted the systematic research in six databases without restrictions on January 24th, 2022. We investigated adult patient with AP, who were treated with PPI compared to conventional therapy. The pooled odds ratios, mean differences, and corresponding 95% confidence intervals were calculated with random effect model. We included six RCTs and three cohort studies, consisting of 28,834 patients. We found a significant decrease in the rate of pancreatic pseudocyst formation in patients who received PPI treatment. PPI use was associated with a higher risk of GI bleeding, however this finding could be due to the patients’ comorbid conditions. We found no significant difference in the rates of 7-day mortality, length of hospital stay, and acute respiratory distress syndrome between the groups. The available data on this topic are limited; therefore, further well designed RCTs are needed to evaluate the potential benefits and adverse effects of PPIs in AP.

## Introduction

The incidence rate of acute pancreatitis (AP) has significantly increased worldwide in the past half century^1^. The reason for this elevation seems to be a combination between the improvement of diagnostic tools, and a rise in the prevalence of AP risk factors such as ageing population, increased alcohol consumption and obesity^1^. On the basis of the arising complications and severity/occurrence of organ failure, AP can be categorized as ‘mild’, ‘moderately severe’ and ‘severe’ according to the revised Atlanta classification system^2^. The overall mortality is around 2–3%, but it can reach 40% in severe cases^1,3,4^.

Proton pump inhibitors (PPIs) are amongst the most frequently prescribed medications^5^. Several nationwide studies showed that the prevalence of PPI use ranged between 7.4 and 15.5% in outpatient settings and from 44.5 to 54.1% in inpatient settings, and their prescription has been constantly increasing over the past years^6–9^. Although their short-term administration is usually without side effects, there is a growing concern regarding their long-term complications (e.g., clostridioides infection, renal diseases, osteoporosis, dementia, small intestine overgrowth and malabsorption)^10^. In AP patients, acid suppression drugs are administered even more frequently according to an international cohort study on 17,422 patients, reaching 23.3% on admission and 86.6% during hospitalization^11^.

Acute gastric mucosal lesions caused by stress are more likely to occur in patients with severe AP^12^, which increase the risk of GI bleeding and ulceration. Therefore, protecting the gastric mucosa seems to be a crucial therapeutic objective. The pillars of the acid secretion suppression are H_2_-receptor inhibitors and PPIs. Theoretically, PPIs could also decrease the pancreatic exocrine secretion by inhibiting the activity of H^+^/K^+^ ATPases within the pancreatic ducts that are similar to the gastric ATPases^13^. Experimental studies had controversial results regarding their capability of reducing pancreatic amylase secretion^14,15^. Moreover, in experimentally induced pancreatitis, pantoprazole reduced inflammation and necrosis^15^. Theoretically, PPI administration can be a good therapeutic option for the protection of the upper GI mucosa and to rest the inflamed pancreas.

The use of PPIs for the treatment of AP can be considered as an off-label indication; however, the usual indications of PPIs may coincide with AP treatment. Even though PPIs are commonly used in clinical practice, current AP guidelines do not specify their administration^16–19^. Two cohort studies reported significantly increased severity among AP cases in which PPIs were prescribed that may be associated with an increased mortality^11,20^. As previous studies had contradictory findings regarding the impact of PPIs on the prognosis of AP patients, our aim was to investigate the associations between PPIs in AP and various clinical outcomes in this systematic review and meta-analysis.

## Results

### Description of included studies

**Figure 1:**
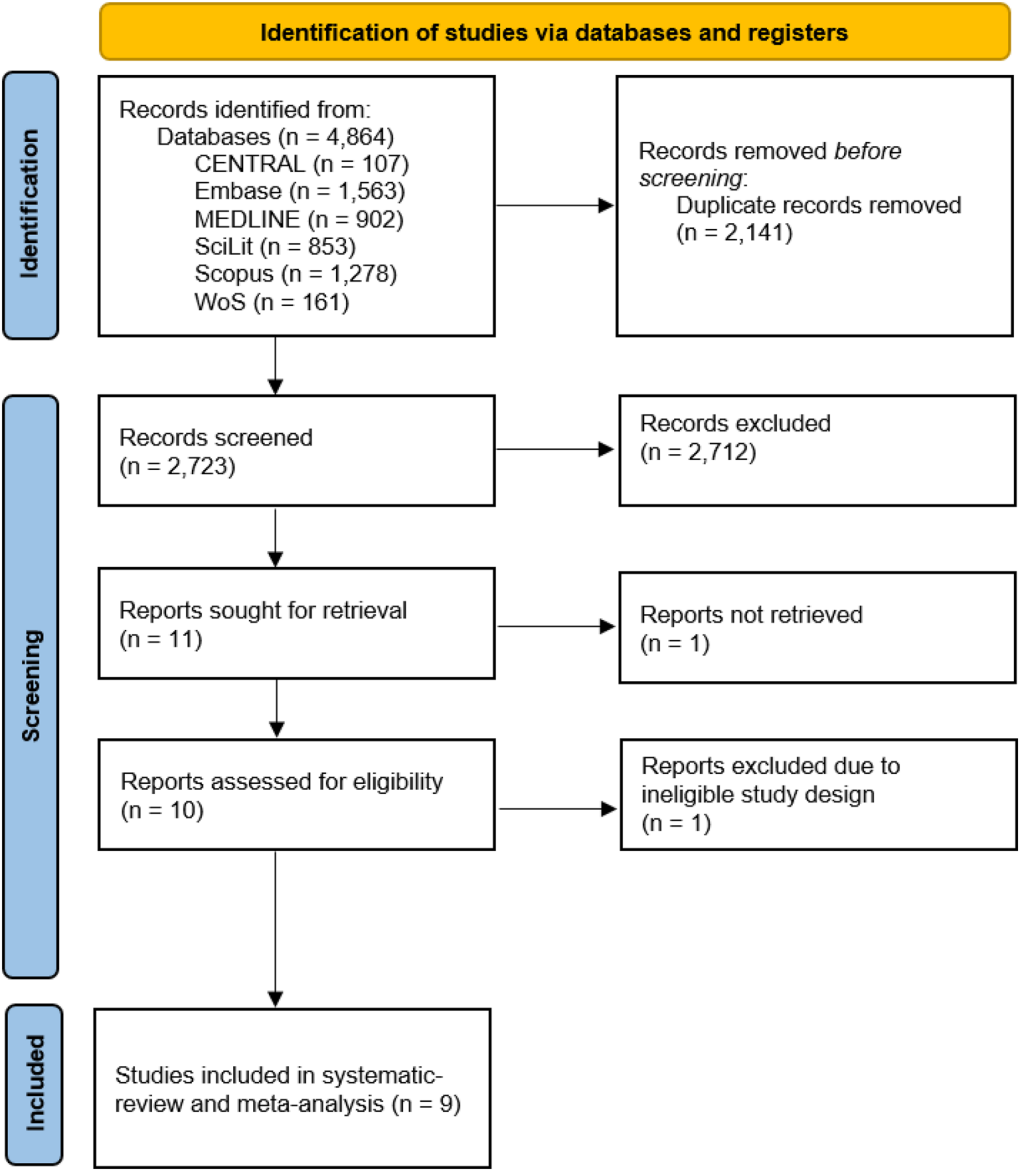
PRISMA flowchart. *CENTRAL* Cochrane Central Register of Controlled Trials, *WoS* Web of Science.

The systematic search resulted in 4864 records. We discarded 2141 records in the manual and automatic duplicate removal process. After the title and abstract, and the full-text selection (Cohen’s kappa 0.95 and 1.00, respectively), we found nine eligible studies to include in the systematic review, comprising 28,834 patients. The detailed identification process and the patient characteristics are summarized in Fig. 1 and Table 1, respectively.

**Table 1:** Baseline characteristics of included articles.

Study	Type	Origin	Sample size (female %)	Mean age ± SD (years)	Acute pancreatitis severity	Experimental group	Experimental group sample size (female %)	Experimental mean age ± SD (years)	Control group	Control group sample size (female %)	Control group mean age ± SD (years)	Outcome
Demcsák^11^	cohort	Inter-national	17,422 (43.6%)	56.5 ± 17.9	All form	Dexlansoprazole, esomeprazole, ilaprazole, lansoprazole, omeprazole, pantoprazole, rabeprazole	12,764 (43.6%)	56.8 ± 17.9	SoC	4658 (44.2%	55.6 ± 17.6	GI bleeding
Hong^22^	RCT	China	96 (53.1%)	NR*	Severe	3 mg somatostatin + 40 mg IV omeprazole q24h for 7d then 3 mg somatostatin + 40 mg IV omeprazole q12h for 7d	48 (54.2%)	NR*	SoC	48 (52.1%)	NR*	ARDS GI bleeding Pancreatic pseudocyst
Ma^23^	RCT	China	45 (40.0%)	45.4 ± NR	Severe	Octreotide 50 mcg/h for 72 h then 25mcg/h for 96 h + esomeprazole 40 mg IV for 7d	24 (33.3%)	44.8 ± 10.6	octreotide 50 mcg/h for 72 h then 25mcg/h for 96 h SoC	21 (47.6%)	46.0 ± 11.7	GI bleeding Laboratory parameters
Ma^26^	RCT	China	66 (34.8%)	45.3 ± NR	Severe	Esomeprazole 40 mg q24h	33 (30.3%)	46.1 ± 11.1	SoC	33 (39.4%)	44.6 ± 9.3	Mortality (7d)
Murata^19^	cohort	Japan	10,400	NR**	Severe	Lansoprazole or omeprazole	3879 (33.1%)	NR**	SoC	6521 (34.8%)	NR**	Mortality (7d)
Wang^25^	RCT	China	160 (46.3%)	63.4 ± NR	Severe	3 mg somatostatin + 40 mg IV esomeprazole q24h for 7d then 6 mg somatostatin + 40 mg IV esomeprazole q12h for 14d	80 (45.0%)	63.4 ± 8.0	SoC	80 (47.5%)	63.3 ± 8.5	LOHS
Xia^20^	RCT	China	140 (34.3%)	42.8 ± NR	Severe	3 mg somatostatin + 40 omeprazole IV q24h for 7d	70 (37.1%)	41.67 ± 22.56	SoC	70 (31.4%)	43.85 ± 19.71	ARDS Mortality (7d) LOHS Pancreatic pseudocyst
Yoo^24^	RCT	South Korea	40 (20.0%)	48.5 ± NR	All form	Pantoprazole IV or PO q12h	20 (15.0%)	49.3 ± 16.5	SoC	20 (25.0%)	47.6 ± 18.3	LOHS
Zhang^21^	cohort	China	858 (37.9%)	56.0 ± NR	All form	Esomeprazole, omeprazole or pantoprazole	336 (45.5%)	56.59 ± 17.17	SoC	174 (47.7%)	55.4 ± 17.03	ARDS LOHS Mortality (7d) Pancreatic pseudocyst

*SD* standard deviation, *RCT* randomized controlled trial, *PPI* proton pump inhibitor, *SoC* standard of care, *NR* not reported, *mg* milligram, mcg microgram, *IV* intravenous, *PO* per os, *q* every, *h* hour, *d* day, *GI* gastrointestinal, *ARDS* acute respiratory distress syndrome, *AKI* acute kidney injury, *LOHS* length of hospital stay.

*Min–max age was reported.

**Age groups were reported.

#### The association between PPIs and complication rates in acute pancreatitis

**Figure 2:**
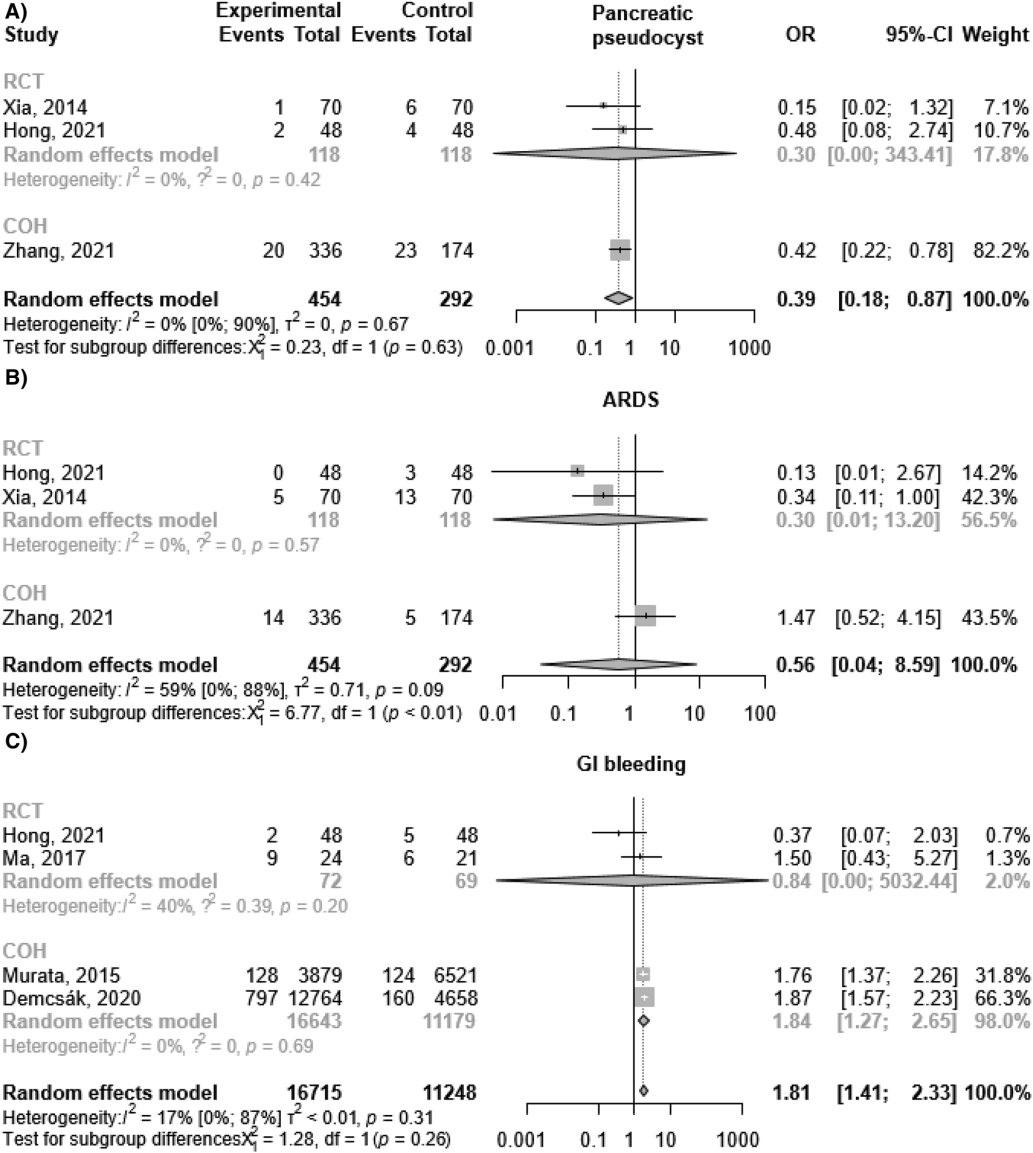
The addition of proton pump inhibitor treatment to standard of care in acute pancreatitis was associated with: (**A**) decreased pancreatic pseudocyst development rate; (**B**) no significant difference regarding development of ARDS; (**C**) increased odds of GI bleeding. *OR* odds ratio, *CI* confidence interval, *RCT* randomized controlled trial, *COH* cohort study, *ARDS* Acute Respiratory Distress Syndrome, *GI* gastrointestinal.

The analysis of the pooled results from three studies^21–23^ including 746 patients showed that in the intervention group the rate of pseudocyst development decreased by 61% compared to the control group [OR 0.39; 95%CI 0.18–0.87; I^2^ = 0%; Fig. 2A]. ARDS incidence was reported in three studies (746 patients), and there was no significant difference between the two groups in this concern [OR 0.56; 95%CI 0.04–8.59; I^2^ = 59%; Fig. 2B]^21–23^. As for GI bleeding, our pooled results from four studies, including 27,963 patients^11,20–24^ revealed increased odds of occurrence in the cases of PPI administration [OR 1.81; 95% CI 1.41–2.33; I^2^ = 17%; Fig. 2C].

#### Length of hospital stay

**Figure 3:**
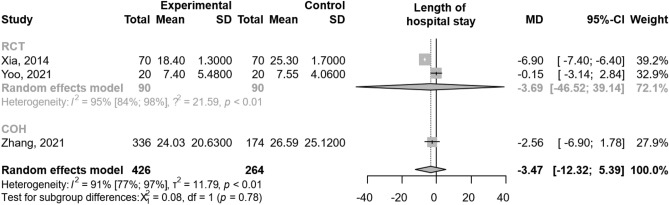
The addition of proton pump inhibitor treatment to standard of care in acute pancreatitis was not associated with a significant change in length of hospital stay by comparison with standard of care alone. *MD* mean difference, *CI* confidence interval, *RCT* randomized controlled trial, *COH* cohort study.

The pooled results from three trials (690 patients)^21,22,25^, including all severity forms of AP, did not show statistically significant difference between the groups regarding the length of hospital stay [MD − 3.47; 95% CI (− 12.32) to 5.39; I^2^ = 91%; Fig. 3]. Wang et al.^26^ has reported on the length of hospital stay; however, we had to exclude the result from the analysis due to a contradiction between the written and graphical results.

#### 7-day mortality

**Figure 4:**
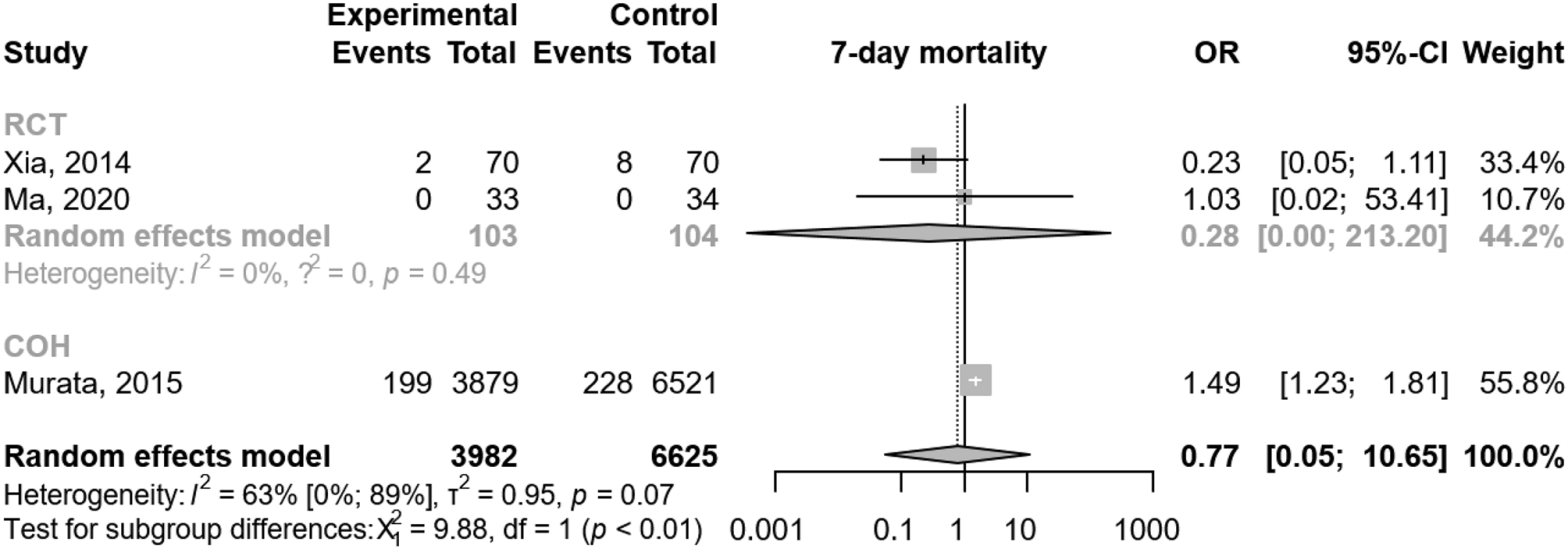
The addition of proton pump inhibitor treatment to standard of care in acute pancreatitis was not associated with a significant change in mortality by comparison with standard of care alone. *OR* odds ratio, *CI* confidence interval, *RCT* randomized controlled trial, *COH* cohort study.

Mortality rates were variously reported across the eligible studies, in terms of moment of evaluation. We were able to perform a meta-analysis for mortality at seven days after diagnosis. In three studies^20,21,27^ including 10,607 patients, there were no significant differences between the experimental and the control group [OR 0.77; 95% CI 0.05–0.10.65; I^2^ = 63%; Fig. 4].

#### Qualitative assessment

Due to insufficient data, we could not perform a meta-analysis for all the outcomes reported across the identified eligible reports.

Murata et al. reported overall mortality and mortality at 14 and 28 days^20^; Zhang et al. reported only in-hospital mortality^22^. Both cohort studies reported similar results, which seem to favour the control group (Table S1).

Two studies reported a decreased rate of acute kidney injury in the PPI group^21,22^. PPI use seems to have a beneficial effect on oxidative, anti- and pro-inflammatory parameters. After the therapy of PPIs combined with somatostatin or octreotide in the intervention group (1) TNFα decreased; (2) pro-inflammatory interleukins (IL-1ß; IL-6; IL-8) and CRP were lower or not significantly different from the control group; (3) anti-inflammatory interleukins (IL-4; IL-10) increased or were similar to the control group (Table S2).

Three studies reported decreased oxidative stress markers (malondialdehyde, lipid hydrogen peroxide and advanced oxidation protein products), chemokines (chemotactic factor protein, fractalkine, neutrophil chemotactic factor); and increased antioxidant indicators (superoxide dismutase, glutathione peroxidase, and catalase)^23,24,26^. Wang et al., Hong and Mi reported that there was decreased intestinal mucosal damage when PPIs were combined with somatostatin in the intervention group, based on the decreased serum levels of D-lactic acid and diamine-oxidase compared to the conventional treatment group^23,26^.

#### Risk of bias and GRADE

Most of the included RCTs were evaluated as having some concerns. Potential biases emerged from the inappropriate reporting of the randomization process, the maintenance of blinding, and the measurement of outcomes. There were no accessible study protocols to investigate the selection of reported results except for the study by Ma et al.^27^ (Figs. S1, S2).

The studies by Demcsák et al. and Murata et al. were well-designed and with low risk of bias in every investigated domain, except for the selection of reported results, where prior protocols were missing. In the cohort study conducted by Zhang, we found a critical level of bias in the selection of the participants: they selected patients by PPI intake after the start of the intervention. Furthermore, we found a moderate risk in the classification of intervention—they defined the intervention group after the start of the intervention, and in the measurement of outcomes. The outcome assessors were probably aware of the invention, yet they reported on strong objective outcomes, which were unlikely to be influenced by knowing the group the patients were assigned to (Fig. S3).

On the basis of the GRADE framework, the evidence level was very low in each investigated outcome (Table S3).

## Discussion

AP research is currently dominated by studies on risk factors for pancreatitis^28–30^, with a declining number of papers on therapeutic options^31^. In this systematic review and meta-analysis, we investigated the association between PPIs addition to conventional therapy compared to conventional therapy alone in patients with AP. PPI use in the treatment of AP is associated with a decreased risk of developing pancreatic pseudocysts. However, there were no significant differences between the two groups in terms of 7-day mortality, length of hospital stay, and ARDS incidence rates. Furthermore, we found an increased risk of bleeding in the PPI group.

Pancreatic pseudocysts in AP are caused by the extravasation of pancreatic fluid. They can have either a spontaneous evolution to resorption in time or progress with complications like rupture, bleeding, and infection^32^. In theory, PPIs cannot just reduce the gastric acid secretion, but also secretin-stimulated bicarbonate secretion^33^. Experimental studies showed contradictory results regarding the inhibition of pancreatic enzyme production. Omeprazole failed to suppress amylase release in isolated pancreatic acini; however, pantoprazole significantly reduced amylase secretion in an experiment with rats^14,15^. One case report showed a decrease in serum amylase level after PPI treatment: a patient had acute necrotizing pancreatitis secondary to 6-mercaptopurine (6-MP) therapy, which was resolved by octreotide therapy. However, when initiating 6-MP again, octreotide with 2 mg/kg/day lansoprazole could decrease amylase to the normal level^34^. Nevertheless, no trials to date have further evaluated the hypothesis. Furthermore, PPI elevates the gastric pH level, thus reducing secretin release, which further decreases pancreatic secretion^33^. Our results suggest that there may be a link between PPI use and the decreased rate of pancreatic pseudocyst formation in AP.

In addition, our analyses showed that PPI treatment during AP does not have a significant effect on 7-day mortality, the length of hospital stay and the complication rate of ARDS, indicating no major benefits from adding them to standard of care.

The accurate incidence of GI bleeding in AP is not documented well; however, the frequency of excessive GI haemorrhagic complications in AP are reported in 1.2% to 14.5% of the cases, leading to an increased mortality^35^. On the basis of results, PPI treatment during AP was associated with increased risk for GI bleeding^11,20,23,24^, which could be due to a variety of pancreatic and non-pancreatic conditions, e.g., history of peptic ulcer, and concomitant anticoagulation. In the prevention of upper GI bleeding, PPIs are indisputable, but they might not be as effective against lower GI bleeding, and might even cause small bowel injury by producing dysbiosis in the GI tract, especially when using concomitant warfarin, NSAIDs, or acetylsalicylic acid^36,37^. None of the included articles reported on concomitant medications. Moreover, the cohort study by Demcsák et al.^11^ showed an association between PPI use and the severity of AP: a significant proportion of patients who were administered acid suppressants had moderate or severe episodes of AP. This finding is also supported by Murata et al.^20^.

In severe AP, the released inflammatory mediators (IL-1ß, IL-6 and TNFα) may induce gut dysbacteriosis, which could be enhanced by the acid suppressive effect of PPIs^38–40^. One RCT including 66 patients, showed a significant increase in duodenal dysbiosis, duodenal bacterial overgrowth, and candida oesophagitis cases when using esomeprazole compared to conventional therapy^27^. Furthermore, the released inflammatory mediators can cause hyperpermeability of the intestinal mucosa, which, together with bacterial overgrowth, could lead to bacteraemia. This effect can further activate pro-inflammatory cytokines, resulting in an enhancement of the inflammatory processes^40^.

On the other hand, PPIs seem to be associated with a decreased pro-inflammatory cytokine release that would disrupt the barrier functions^41^. Two studies showed that the serum levels of d-lactic acid and diamine oxidase, which rarely get absorbed from the GI tract in physiological conditions, were lower in the intervention group (which in this case included patients treated with PPI—somatostatin association) compared to the control group, and suggesting a protective effect on the intestinal barrier function^23,26^. However, somatostatin might also have a protective role in the sepsis-induced gut barrier dysfunction according to an animal model study^42^; therefore, combining PPI and somatostatin may have an enhanced protective effect. Moreover, PPIs may show scavenging properties for reactive oxygen species^41^. However, somatostatin can express antioxidant effects and decrease cytokine levels; thus, it could also contribute to the anti-inflammatory effect, when administered in combination with PPIs^23^. The mechanisms behind these effects have not yet been completely described, further investigations are needed.

PPIs are among the most overused medications, they are generally prescribed without any specific indication^5^. Patients with AP received some kind of acid suppressive drugs in 23.3% of cases on admittance to the hospital, 86.6% of the patients received them during hospitalization, and 57.6% when they were discharged from hospital^11^. The prophylactic use of PPIs for stress ulcer prophylaxis in individuals with AP is an off-label indication. PPIs are regarded as safe medications, with a safe adverse effect profile; however, there are possible adverse reactions in the long-term. Drug-induced AP is responsible for 2–5% of the AP cases^43^, and PPIs are rarely associated with its onset^44–48^. Patients who have gastrointestinal reflux disease, peptic ulcer, dyspepsia or had prior GI bleeding are likely to be prescribed PPIs, even in an elevated dose. However, the inappropriate use of PPIs exposes the patient to harm, which could be prevented.

Even though PPIs are widely used during the treatment of AP, this is the first meta-analysis assessing their association with various AP related clinical outcomes. However, several limitations should be emphasized. There is a low number of trials available in the topic, and in many cases they have a moderate to high risk of bias, especially regarding randomization process, deviations from intended intervention, selection of reported results and outcome measurement. In the analyzed trials, different PPIs were used. Although the main mechanism of action of these drugs is common, their activity profiles are different which may influence their clinical effect in AP. For example, omeprazole, in contrast to pantoprazole does not inhibit amylase release from isolated pancreatic acini^49^. Furthermore, there is no information about the initiation and the duration of PPI therapy in report of AP onset, and follow-up times. Moreover, due to their small sample sizes, the RCTs were assigned low weights in the pooled results by comparison with the cohort studies, which can impact the overall effect measurement. This is important as they reported opposite results regarding outcomes like mortality and GI bleeding. The analysis of RCT data alone resulted in different results in case of these two outcomes: no significant differences could be detected between the two treatment groups.

Some of the results were associated with high heterogeneity that can be caused by the variability in the methods across the eligible studies—some of them included only severe AP cases; and standard of care differed across the studies—some included somatostatin analogue therapy although it is not recommended in the current guidelines. Somatostatin and, in theory, PPIs decrease the pancreatic exocrine function, thus they might have additional effects. However, there is no evidence supporting this assumption^33,50^.

Translating scientific results to the daily practice is crucially important^51,52^. The general use of PPIs is not recommended for AP treatment, only certain comorbidities (e.g., peptic ulcer, GERD, and Barret’s esophagus), can justify their use. However, on the basis of our results, patients who have a higher risk of developing pancreatic pseudocysts such as patients who smoke or have increased alcohol consumption, or have chronic pancreatitis, may benefit from the addition of a PPI to the conventional therapy^53^.

Well-designed, RCTS with larger sample sizes are needed to confirm our results. The evaluated outcomes should be clearly defined, and efficacy assessment should be focused on objective parameters, like measurement of inflammatory markers or complication rate. Moreover, comorbidities and concomitant treatments for other diseases should be mentioned, and if possible, their impact should be assessed a multivariate analysis. For cohort analyses, the report of PPI indication would be essential, as our findings regarding the association of PPI treatment with increased risk of GI bleeding is probably artefactual. Possibly, our finding is related to the fact that PPIs represent the treatment for upper GI bleeding.

## Conclusion

Our meta-analysis pointed out that even though PPI use in AP treatment reduced the rates of pancreatic pseudocyst formation, it did not show significant effects on other outcomes. PPIs should be recommended only as an addition to the standard of care, if there is a relevant comorbidity or a higher risk for GI bleeding or developing pancreatic pseudocyst. There is a need for well-designed, RCTs to determine which populations would benefit most from PPI treatment during AP, and most importantly, which are the benefits and drawbacks of PPI use in this disease.

## Methods

### Search and selection strategy

The systematic review and meta-analysis were conducted by the recommendations of the Cochrane collaboration^54^, and the findings are reported by the Preferred Reporting Items for Systematic Reviews and Meta-Analyses (PRISMA) 2020 statement^55^. The review protocol was registered in the International Prospective Register of Systematic Reviews (PROSPERO) database (CRD42022303136).

We used the PICO (Patient/Population, Intervention, Comparison and Outcomes) framework to formulate the research question, and to define eligibility criteria. We investigated adult patients with AP (P), who were treated with PPIs in addition to standard of care (I). The control group did not receive PPI (C), and we investigated outcomes (O) including mortality, length of hospital stay, complications, and change in laboratory parameters. As somatostatin analogues have no proven beneficial effect in the management of AP^56^, the current AP guidelines do not recommend their use^16,17,19^. We also included studies in which combinations of somatostatin analogues and PPIs were used in the intervention group. As for study design, randomized controlled trials (RCTs) and cohort studies were eligible.

The systematic search was performed in six databases (Cochrane Central Register of Controlled Trials, Embase, MEDLINE, Scilit, Scopus, Web of Science) on the 24th of January 2022. The detailed search strategy is found in the supplementary material. We did not use restrictions or filter options during the search. The reference lists of the identified eligible studies were screened for further reports.

The results of the database searches were exported to a citation manager (EndNote X9; Clarivate Analytics, Philadelphia, PA, USA), where automatic and manual duplicate removal was performed by ILH. Two independent authors (ILH and DK) did the abstract and title, and the subsequent full-text selection according to the above-mentioned eligibility criteria. Cohen’s kappa coefficient was calculated for inter-rater reliability evaluation at each selection step. In case of a disagreement, a third author (DCs) made the final decision.

Non-English articles were translated using Google Translate® (Google LLC, Mountain View, CA, USA).

### Data extraction

Two independent authors (ILH and DK) extracted the following data from each eligible study: first author, publication year, study origin, study design, number of patients (sample size), gender distribution, mean age, the severity of disease, applied medications and dosages, duration of the therapy and outcomes according to previously mentioned PICO framework. The data was collected in Microsoft Excel (Microsoft, Office 365, Redmond, WA, USA). To extract data available in graphical format only, we used a dedicated software (Plot Digitizer 2.6.8, 2015).

### Statistics

Odds ratios (OR) with 95% confidence interval (CI) were calculated as an effect size measure for categorical outcomes (rates of acute respiratory distress syndrome (ARDS), pseudocyst formation, 7-day mortality, and GI-haemorrhage) using the total number of patients and the number of events in each group as reported in each study. For continuous outcomes (length of hospital stay), the mean difference between the groups and the corresponding 95% CI was calculated to measure the effect size using the means and standard deviations reported across the eligible articles.

Due to the anticipated considerable between-study heterogeneity, random-effects model was used to pool effect sizes. Pooled OR was calculated by the Mantel–Haenszel method^57–59^. The exact Mantel–Haenszel method (without continuity correction) was used to handle zero cell counts^60,61^. Inverse variance weighting method was used to calculate the pooled mean difference. For all outcomes, a Hartung–Knapp adjustment^62,63^ was used to ensure a conservative estimation of the effect size CIs. For continuous outcomes the restricted maximum-likelihood estimator and for OR measures the Paule–Mandel method^64^ were applied to estimate the variance with the Q profile method for CI^65^.

Between-study heterogeneity was described by means of Cochrane Q test, and the Higgins and Thompson’s I^2^ statistics^66^. To estimate the heterogeneity variance measure τ^2^ was used. Forest plots were used to graphically summarize the results. In case of mean difference effect size measures, the CI of individual study was calculated based on the t-distribution. Outlier and influence analyses were carried out by conducting a leave-one-out analysis, and comparing the results of the leave-one-out runs and the original meta-analysis^67,68^. All statistical analyses were performed with R [v4.1.2] using the meta [5.2.0] package.

### Risk of bias and certainty of evidence assessment

Two authors (ILH and DK) independently evaluated the risk of bias for each included study, and a third author (DCs) resolved the disagreements. The revised Cochrane Risk-of-Bias tool (RoB2)^69^ was used to assess the bias in RCTs, whereas the cohort studies were evaluated by Risk Of Bias in Non-randomized Studies-of Intervention (ROBINS-I) tool^70^.

The framework Grading of Recommendations, Assessment, Development and Evaluations (GRADE) and the corresponding tool^71^ were used to evaluate each outcome for certainty of evidence. Each outcome was rated for risk of bias, inconsistency, indirectness, imprecision, publication bias, and the presence of a large effect, dose-dependent response, and plausible confounders as ‘not serious’, ‘serious’, or ‘very serious’. The final certainty of the evidence was categorized as ‘very low’, ‘low’, ‘moderate’, or ‘high’.

## Supplementary Information

Supplementary Information 1.

Supplementary Information 2.

## Data Availability

Data used for the analysis are available from the corresponding author upon reasonable request. The datasets used in this study can be found in the full-text articles included in the systematic review and meta-analysis.

## References

[1] Iannuzzi JP (2022). Global incidence of acute pancreatitis is increasing over time: A systematic review and meta-analysis. Gastroenterology.

[2] Banks PA (2013). Classification of acute pancreatitis—2012: Revision of the Atlanta classification and definitions by international consensus. Gut.

[3] Kui B (2022). EASY-APP: An artificial intelligence model and application for early and easy prediction of severity in acute pancreatitis. Clin. Transl. Med..

[4] Parniczky A (2016). Prospective, multicentre, nationwide clinical data from 600 cases of acute pancreatitis. PLoS ONE.

[5] Fuentes AV, Pineda MD, Venkata KCN (2018). Comprehension of top 200 prescribed drugs in the US as a resource for pharmacy teaching, training and practice. Pharmacy (Basel).

[6] Daniels B, Pearson SA, Buckley NA, Bruno C, Zoega H (2020). Long-term use of proton-pump inhibitors: Whole-of-population patterns in Australia 2013–2016. Therap. Adv. Gastroenterol..

[7] Pottegard A (2016). Use of proton-pump inhibitors among adults: A Danish nationwide drug utilization study. Therap. Adv. Gastroenterol..

[8] Halfdanarson OO (2018). Proton-pump inhibitors among adults: A nationwide drug-utilization study. Therap. Adv. Gastroenterol..

[9] Matuz M (2020). Use of proton pump inhibitors in Hungary: Mixed-method study to reveal scale and characteristics. Front. Pharmacol..

[10] Fossmark R, Martinsen TC, Waldum HL (2019). Adverse effects of proton pump inhibitors-evidence and plausibility. Int. J. Mol. Sci..

[11] Demcsak A (2020). Acid suppression therapy, gastrointestinal bleeding and infection in acute pancreatitis—An international cohort study. Pancreatology.

[12] Dang SC (2015). Are gastric mucosal macrophages responsible for gastric injury in acute pancreatitis?. World J. Gastroenterol..

[13] Wang J (2015). Proton pump inhibitors inhibit pancreatic secretion: role of gastric and non-gastric H+/K+-ATPases. PLoS ONE.

[14] Cai J, Zhou W, Luo HS, Peng LV (2007). Effect of proton pump inhibitor on amylase release from isolated pancreatic acini. In Vitro Cell Dev. Biol. Anim..

[15] Hackert T (2010). Effects of pantoprazole in experimental acute pancreatitis. Life Sci..

[16] Crockett SD (2018). American Gastroenterological Association Institute Guideline on initial management of acute pancreatitis. Gastroenterology.

[17] Leppaniemi A (2019). 2019 WSES guidelines for the management of severe acute pancreatitis. World J. Emerg. Surg..

[18] Chinese Pancreatic Surgery Association, Chinese Society of Surgery, Chinese Medical Association (2021). Guidelines for diagnosis and treatment of acute pancreatitis in China. Zhonghua Wai Ke Za Zhi.

[19] Tenner S, Baillie J, DeWitt J, Vege SS, American College of Gastroenterology (2013). American College of Gastroenterology guideline: Management of acute pancreatitis. Am. J. Gastroenterol..

[20] Murata A, Ohtani M, Muramatsu K, Matsuda S (2015). Effects of proton pump inhibitor on outcomes of patients with severe acute pancreatitis based on a national administrative database. Pancreatology.

[21] Xia YX, Liu XZ, Zhang XD, Shang PJ, Guo J (2014). Efficacy and safety of omeprazole combined with somatostatin in treatment of severe acute pancreatitis. World Chin. J. Digestol..

[22] Zhang SY (2021). Proton pump inhibitors were associated with reduced pseudocysts in acute pancreatitis: A multicenter cohort study. Front. Pharmacol..

[23] Hong J, Mi J (2021). The effect of somatostatin combined with omeprazole on patients with severe acute pancreatitis. Farmacia.

[24] Ma X (2017). Effect of proton pump inhibitors on severe acute pancreatitis—A prospective randomized trial. J. Sichuan Univ. Med. Sci. Ed..

[25] Yoo JH (2012). Effect of proton pump inhibitor in patients with acute pancreatitis—Pilot study. Korean J. Gastroenterol..

[26] Wang K, Lv S (2020). Effects of esomeprazole sodium combined with somatostatin on serum inflammatory indexes and intestinal barrier function in patients with severe acute pancreatitis. Int. J. Clin. Exp. Med..

[27] Ma X (2020). The impacts of acid suppression on duodenal microbiota during the early phase of severe acute pancreatitis. Sci. Rep..

[28] Szentesi A (2022). Alcohol consumption and smoking dose-dependently and synergistically worsen local pancreas damage. Gut..

[29] Ocskay K (2021). Hypoalbuminemia affects one third of acute pancreatitis patients and is independently associated with severity and mortality. Sci. Rep..

[30] Nagy A (2021). Glucose levels show independent and dose-dependent association with worsening acute pancreatitis outcomes: Post-hoc analysis of a prospective, international cohort of 2250 acute pancreatitis cases. Pancreatology.

[31] Nagy R (2022). In-hospital patient education markedly reduces alcohol consumption after alcohol-induced acute pancreatitis. Nutrients.

[32] Habashi S, Draganov PV (2009). Pancreatic pseudocyst. World J. Gastroenterol..

[33] Chey WY, Chang TM (2003). Secretin, 100 years later. J. Gastroenterol..

[34] Fettah A, Yarali N, Bayram C, Kirsaclioglu CT, Tunc B (2014). Proton pump inhibitor therapy in chemotherapy-induced pancreatitis. J. Pediatr. Hematol. Oncol..

[35] Rana SS (2015). Gastrointestinal bleeding in acute pancreatitis: Etiology, clinical features, risk factors and outcome. Trop. Gastroenterol..

[36] Tang J (2021). A study of proton pump inhibitors and other risk factors in warfarin-associated gastrointestinal bleeding. Cureus.

[37] Lue A, Lanas A (2016). Protons pump inhibitor treatment and lower gastrointestinal bleeding: Balancing risks and benefits. World J. Gastroenterol..

[38] Su SY, Tang QQ (2021). Altered intestinal microflora and barrier injury in severe acute pancreatitis can be changed by zinc. Int. J. Med. Sci..

[39] Ge P (2020). Intestinal barrier damage, systemic inflammatory response syndrome, and acute lung injury: A troublesome trio for acute pancreatitis. Biomed. Pharmacother..

[40] Watanabe T, Kudo M, Strober W (2017). Immunopathogenesis of pancreatitis. Mucosal Immunol..

[41] Kedika RR, Souza RF, Spechler SJ (2009). Potential anti-inflammatory effects of proton pump inhibitors: A review and discussion of the clinical implications. Dig. Dis. Sci..

[42] Xu X (2020). Protective role of somatostatin in sepsis-induced intestinal barrier dysfunction through inhibiting the activation of NF-kappaB pathway. Gastroenterol. Res. Pract..

[43] Meczker A (2020). Analysis of 1060 cases of drug-induced acute pancreatitis. Gastroenterology.

[44] Kathi P, Smith S, Rabadi R, Thammineni N, Mutchnick M (2020). Omeprazole-associated necrotizing pancreatitis. Am. J. Ther..

[45] Youssef SS, Iskandar SB, Scruggs J, Roy TM (2005). Acute pancreatitis associated with omeprazole. Int. J. Clin. Pharmacol. Ther..

[46] Das S (2012). Oral pantoprazole-induced acute pancreatitis in an 11-year-old child. Ther. Drug Monit..

[47] Murtaza G, Khalid MF, Mungo NA (2018). Recurrent pantoprazole-associated pancreatitis. Am. J. Ther..

[48] Ocal S (2014). Lansoprazole-induced acute pancreatitis. Turk. J. Gastroenterol..

[49] Scarpignato C (2016). Effective and safe proton pump inhibitor therapy in acid-related diseases—A position paper addressing benefits and potential harms of acid suppression. BMC Med..

[50] Banks PA, Freeman ML, Practice Parameters Committee of the American College of Gastroenterology (2006). Practice guidelines in acute pancreatitis. Am. J. Gastroenterol..

[51] Hegyi P, Eross B, Izbeki F, Parniczky A, Szentesi A (2021). Accelerating the translational medicine cycle: The Academia Europaea pilot. Nat. Med..

[52] Hegyi P (2020). Academia Europaea position paper on translational medicine: The cycle model for translating scientific results into community benefits. J. Clin. Med..

[53] Szako L (2021). Early occurrence of pseudocysts in acute pancreatitis—A multicenter international cohort analysis of 2275 cases. Pancreatology..

[54] Higgins, J. P. T. *et al*. *Cochrane Handbook for Systematic Reviews of Interventions* (Cochrane, 2021).

[55] Page MJ (2021). The PRISMA 2020 statement: An updated guideline for reporting systematic reviews. BMJ.

[56] Moggia E (2017). Pharmacological interventions for acute pancreatitis. Cochrane Database Syst. Rev..

[57] Mantel N, Haenszel W (1959). Statistical aspects of the analysis of data from retrospective studies of disease. J. Natl. Cancer Inst..

[58] Robins J, Greenland S, Breslow NE (1986). A general estimator for the variance of the Mantel–Haenszel odds ratio. Am. J. Epidemiol..

[59] Thompson SG, Turner RM, Warn DE (2001). Multilevel models for meta-analysis, and their application to absolute risk differences. Stat. Methods Med. Res..

[60] Cooper H, Hedges LV, Valentine JC (2019). The Handbook of Research Synthesis and Meta-Analysis.

[61] Sweeting MJ, Sutton AJ, Lambert PC (2004). What to add to nothing? Use and avoidance of continuity corrections in meta-analysis of sparse data. Stat. Med..

[62] Knapp G, Hartung J (2003). Improved tests for a random effects meta-regression with a single covariate. Stat. Med..

[63] IntHout J, Ioannidis JPA, Borm GF (2014). The Hartung–Knapp–Sidik–Jonkman method for random effects meta-analysis is straightforward and considerably outperforms the standard DerSimonian–Laird method. BMC Med. Res. Methodol..

[64] Paule RC, Mandel J (1982). Consensus values and weighting factors. J. Res. Natl. Bur Stand.

[65] Veroniki AA (2016). Methods to estimate the between-study variance and its uncertainty in meta-analysis. Res. Synth. Methods.

[66] Higgins JP, Thompson SG (2002). Quantifying heterogeneity in a meta-analysis. Stat. Med..

[67] Harrer M, Cuijpers P, Furukawa TA, Ebert DD (2021). Doing Meta-Analysis with R: A Hands-on Guide.

[68] Viechtbauer W, Cheung MWL (2010). Outlier and influence diagnostics for meta-analysis. Res. Synth. Methods.

[69] Sterne JAC (2019). RoB 2: A revised tool for assessing risk of bias in randomised trials. BMJ.

[70] Sterne JA (2016). ROBINS-I: A tool for assessing risk of bias in non-randomised studies of interventions. BMJ.

[71] *GRADEpro GDT: GRADEpro Guideline Development Tool*. www.gradepro.org (2021).

