# Supplementary material for: No evidence for the benefit of PPIs in the treatment of acute pancreatitis: a systematic review and meta-analysis
Source: Sci Rep. 2023 Feb 16;13:2791. doi: 10.1038/s41598-023-29939-5 (PMC9935541; doi:10.1038/s41598-023-29939-5)
Supplement: Supplementary file 2 — Supplementary Information 2. [file 41598_2023_29939_MOESM2_ESM.docx]

**No evidence for the benefit of PPIs in the treatment of acute pancreatitis: a systematic review and meta-analysis**

István László Horváth^1,2^, Stefania Bunduc^1,3,4,5^, Dénes Kleiner^1,2^, Alexandra Demcsák^6^, Bence Szabó^1^, Péter Hegyi^1,3,7^, Dezső Csupor^1,7,8*^

^1^ Centre for Translational Medicine, Semmelweis University, 1085 Budapest, Üllői út 26, Hungary

^2^ University Pharmacy Department of Pharmacy Administration, 1092 Budapest, Hőgyes Endre utca 7-9., Hungary

^3^ Division of Pancreatic Diseases, Heart and Vascular Center, Semmelweis University, 1085 Budapest, Baross út 22-24, Hungary

^4^ Carol Davila University of Medicine and Pharmacy, 020021 Bucharest, Dionisie Lupu street 37, Romania

^5^ Fundeni Clinical Institute, 022328 Bucharest, Fundeni street 258, Romania

^6^ Department of Surgery, University of California Los Angeles, Los Angeles, 675 Charles E Young Dr S MRL 2220, California 90095, USA

^7^ Institute for Translational Medicine, Medical School, University of Pécs, 7624 Pécs, Szigeti út 12, Hungary

^8^ Institute of Clinical Pharmacy, University of Szeged, 6725 Szeged, Szikra utca 8, Hungary

**Search strategy:**

We used the following serchterm in the systematic database search:

*pancreatitis AND (PPI OR PPIs OR (proton pump inhibitor*) OR omeprazole OR pantoprazole OR lansoprazole OR rabeprazole OR esomeprazole OR dexlansoprazole OR dexrabeprazole OR vonoprazan OR tegoprazan)*

| Study | Experimental sample size | Experimental event | Control sample size | Control event |
| --- | --- | --- | --- | --- |
|  | Mortality within 14 days | | | |
| Murata, 2015 | 3879 | 199 (5,13%) | 6521 | 228 (3,50%) |
|  | Mortality within 28 days | | | |
| Murata, 2015 | 3879 | 272 (7,01%) | 6521 | 292 (4,48%) |
|  | Mortality overall | | | |
| Murata, 2015 | 3879 | 360 (9,28%) | 6521 | 340 (5,21%) |
|  | Mortality in-hospital | | | |
| Zhang, 2021 | 336 | 3 (0,89%) | 174 | 0 (0,00% |

**Table S1.** Mortality results as reported across the included studies.

| Study | Experimental sample size | Experimental before value ± SD | Experimental after value ± SD | Control sample size | Control before value ± SD | Control after value ± SD |
| --- | --- | --- | --- | --- | --- | --- |
|  | TNFα | | | | | |
| Hong, 2021 (pg/mL) | 48 | 34,52 ± 6,51 | 13,20 ± 3,80 | 48 | 34,15 ± 6,33 | 17,35 ± 3,44 |
| Ma, 2017 (ug/mL) | 24 | 9,47 ± 0,76 | 10,59 ± 0,76 | 21 | 10,02 ± 0,96 | 13,26 ± 2,12 |
| Wang, 2020 (pg/mL) | 80 | 144,80 ± 23,12 | 62,18 ± 12,46 | 80 | 150,89 ± 21,35 | 95,83 ± 11,57 |
|  | IL-1ß | | | | | |
| Hong, 2021 (pg/mL) | 48 | 25,70 ± 3,26 | 4,19 ± 1,09 | 48 | 26,23 ± 3,80 | 9,97 ± 1,09 |
|  | IL-6 | | | | | |
| Ma, 2017 (pg/mL) | 24 | 201,69 ± 57,58 | 39,28 ± 6,14 | 21 | 152,34 ± 27,83 | 38,90 ± 5,51 |
| Wang, 2020 (pg/mL) | 80 | 216,01 ± 24,12 | 95,39 ± 10,96 | 80 | 214,91 ± 24,12 | 137,06 ± 9,87 |
|  | IL-8 | | | | | |
| Ma, 2017  (pg/mL) | 24 | 66,80 ± 25,01 | 20,83 ± 2,69 | 21 | 59,32 ± 25,01 | 15,00 ± 1,66 |
| Hong, 2021 (pg/mL) | 48 | 19,94 ± 1,99 | 4,95 ± 0,91 | 48 | 19,58 ± 2,53 | 9,28 ± 1,27 |
|  | CRP | | | | | |
| Ma, 2017 (mg/mL) | 24 | 249,36 ± 26,68 | 110,21 ± 13,05 | 21 | 244,28 ± 27,31 | 116,91 ± 14,91 |
| Hong, 2021 (mg/L) | 48 | 35,39 ± 5,25 | 10,99 ± 2,90 | 48 | 35,75 ± 5,25 | 19,67 ± 3,08 |
|  | IL-4 | | | | | |
| Wang, 2020 (pg/mL) | 80 | 76,21 ± 7,79 | 106,79 ± 13,18 | 80 | 74,15 ± 6,01 | 88,60 ± 15,56 |
|  | IL-10 | | | | | |
| Wang, 2020 (pg/mL) | 80 | 36,25 ± 9,00 | 84,75 ± 13,78 | 80 | 34,79 ± 9,60 | 53,42 ± 10,78 |

**Table S2**. Summary for reported results of the pro- and anti-inflammatory parameters.

**Figure S1.** The summary for risk of bias assessment of randomized controlled trials via RoB2 tool.


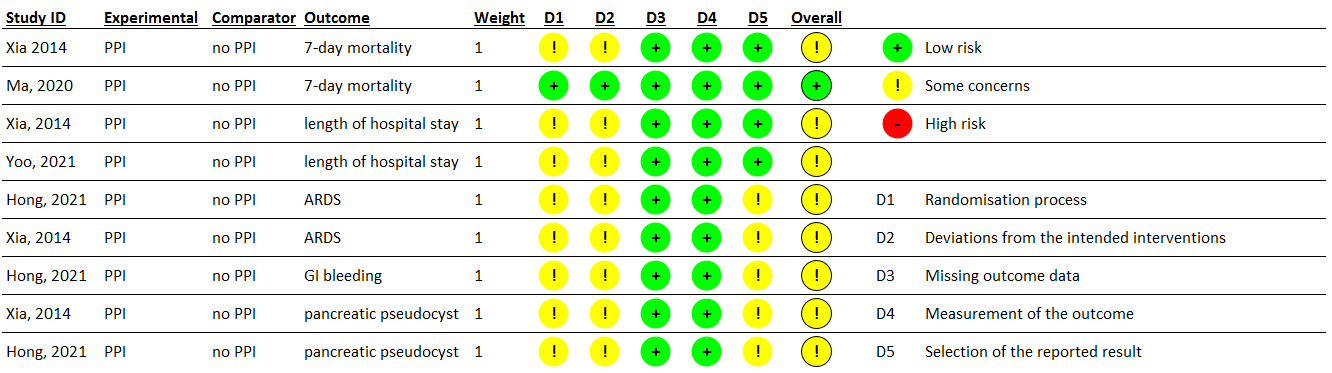


**Figure S2.** The result of risk of bias assessment for randomized controlled trials in detail for each outcome via RoB2 tool.


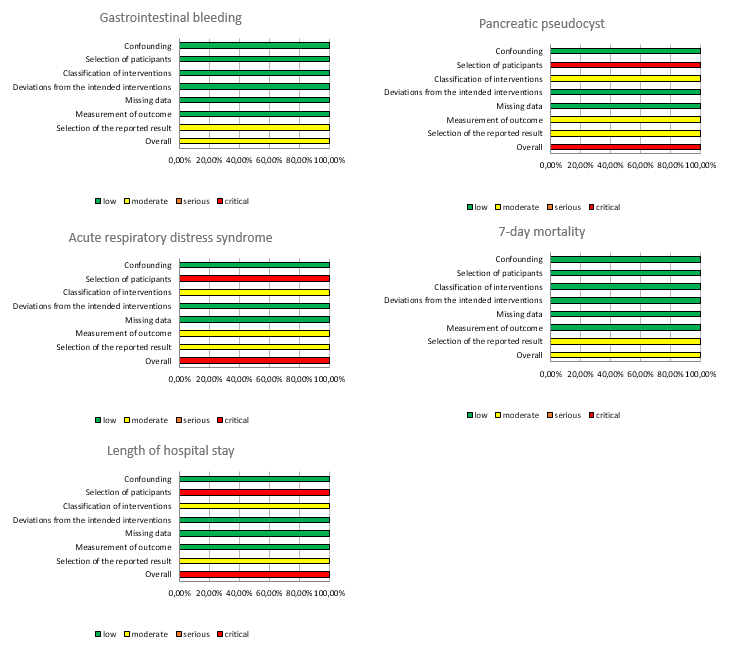

**Figure S3.** The results of risk of bias assessment for cohort studies for each outcome via ROBINS-I tool

| **Certainty assessment** | | | | | | | **№ of patients** | | **Effect** | | **Certainty** | **Importance** |
| --- | --- | --- | --- | --- | --- | --- | --- | --- | --- | --- | --- | --- |
| **№ of studies** | **Study design** | **Risk of bias** | **Inconsistency** | **Indirectness** | **Imprecision** | **Other considerations** | **PPI** | **conventional** | **Relative (95% CI)** | **Absolute (95% CI)** |  |  |
| **GI bleeding** | | | | | | | | | | | | |
| 4 | 2 RCT  2 Cohort | not serious | serious^a^ | serious^b^ | not serious | none | 936/16715 (5.6%) | 295/11248 (2.6%) | **OR 1.81** (1.41 to 2.33) | **20 more per 1 000** (from 10 more to 33 more) | ⨁◯◯◯ Very low | IMPORTANT |
| **Pancreatic pseudocyst** | | | | | | | | | | | | |
| 3 | 2 RCT  1 Cohort | serious^c^ | not serious | serious^b^ | not serious | none | 23/454 (5.1%) | 33/292 (11.3%) | **OR 0.39** (0.18 to 0.87) | **66 fewer per 1 000** (from 91 fewer to 13 fewer) | ⨁◯◯◯ Very low | IMPORTANT |
| **Acute Respiratory Distress Syndrome** | | | | | | | | | | | | |
| 3 | 2 RCT  1 Cohort | not serious | not serious | serious^b^ | not serious | none | 19/454 (4.2%) | 21/292 (7.2%) | **OR 0.56** (0.04 to 8.59) | **30 fewer per 1 000** (from 69 fewer to 328 more) | ⨁◯◯◯ Very low | IMPORTANT |
| **7-day mortality** | | | | | | | | | | | | |
| 3 | 2 RCT  1 Cohort | not serious | serious^d^ | serious^b^ | not serious | none | 201/3982 (5.0%) | 236/6625 (3.6%) | **OR 0.77** (0.05 to 10.65) | **8 fewer per 1 000** (from 34 fewer to 247 more) | ⨁◯◯◯ Very low | IMPORTANT |
| **Length of hospital stay** | | | | | | | | | | | | |
| 3 | 2 RCT  1 Cohort | not serious | serious^e^ | serious^b^ | not serious | none | 426 | 264 | - | MD **3.47 day lower** (12.32 lower to 5.39 higher) | ⨁◯◯◯ Very low | IMPORTANT |

**Table S3.** The summary of level of evidence grading via GRADE tool.

Abbreviations: CI: confidence interval; MD: mean difference; OR: odds ratio; a: RCT showed opposite result compared to cohorts; b: RCT used additional somatostatin therapy in the intervention group; c: Cohort included patients after the start of intervention; d: Xia, 2014 had fourth of the OR compared to the other result
